# Characterization of Two Transposable Elements and an Ultra-Conserved Element Isolated in the Genome of *Zootoca vivipara* (Squamata, Lacertidae)

**DOI:** 10.3390/life13030637

**Published:** 2023-02-24

**Authors:** Marcello Mezzasalma, Teresa Capriglione, Larissa Kupriyanova, Gaetano Odierna, Maria Michela Pallotta, Agnese Petraccioli, Orfeo Picariello, Fabio M. Guarino

**Affiliations:** 1 Department of Biology, Ecology and Earth Science, University of Calabria, Via P. Bucci 4/B, 87036 Rende, Italy; 2 Department of Biology, University of Naples Federico II, Via Cinthia 26, 80126 Naples, Italy; 3 Zoological Institute, Russian Academy of Sciences, 190121 St. Petersburg, Russia; 4 Institute for Biomedical Technologies, National Research Council, 56124 Pisa, Italy

**Keywords:** amniotes, DNA transposons, evolutionarily conserved elements, SINEs, Tc1/Mariner, squamates, reptiles

## Abstract

Transposable elements (TEs) constitute a considerable fraction of eukaryote genomes representing a major source of genetic variability. We describe two DNA sequences isolated in the lizard *Zootoca vivipara*, here named Zv516 and Zv817. Both sequences are single-copy nuclear sequences, including a truncation of two transposable elements (TEs), SINE Squam1 in Zv516 and a Tc1/Mariner-like DNA transposon in Zv817. FISH analyses with Zv516 showed the occurrence of interspersed signals of the SINE Squam1 sequence on all chromosomes of *Z. vivipara* and quantitative dot blot indicated that this TE is present with about 4700 copies in the *Z. vivipara* genome. FISH and dot blot with Zv817 did not produce clear hybridization signals. Bioinformatic analysis showed the presence of active SINE Squam 1 copies in the genome of different lacertids, in different mRNAs, and intronic and coding regions of various genes. The Tc1/Mariner-like DNA transposon occurs in all reptiles, excluding *Sphenodon* and Archosauria. Zv817 includes a trait of 284 bp, representing an amniote ultra-conserved element (UCE). Using amniote UCE homologous sequences from available whole genome sequences of major amniote taxonomic groups, we performed a phylogenetic analysis which retrieved Prototheria as the sister group of Metatheria and Eutheria. Within diapsids, Testudines are the sister group to Aves + Crocodylia (Archosauria), and Sphenodon is the sister group to Squamata. Furthermore, large trait regions flanking the UCE are conserved at family level.

## 1. Introduction

Transposable elements (TEs) constitute a considerable fraction of eukaryote genomes. They can move and amplify within the host DNA, thus representing a major source of genetic variability [1]. The transposition activity of TEs may have a profound effect on the structure and function of the host genome, including the modification and duplication of genetic information and gene expression [1]. Studies on these elements are particularly important for better understanding the mechanisms involved in genome evolution (see, e.g., [2]). TE-induced alterations can adversely affect the host DNA, but evidence suggests that they can also provide beneficial adaptive genetic alterations to species and populations [3,4]. TEs are subdivided into two main categories: Class I TEs (retrotransposons), which transpose through the reverse transcription of an RNA intermediate, and Class II TEs (DNA transposons), which can move within the host genome via a single or double-stranded DNA intermediate [5]. The nature, genomic amount, and level of activity of the various families of TEs may vary widely in distinct vertebrate taxa [5]. Although recent studies have greatly improved our knowledge of vertebrate TE genomic landscapes, this information is mostly based on studies on mammals and birds, while data on other taxa are still very limited by comparison. With over 11,700 described species [6], squamate reptiles represent a major amniote taxonomic group, but their TE content and variability, as well as other related genomic features remain poorly explored [7].

Ultra-conserved elements (UCEs) are genomic regions of about 200 bp which are highly conserved in at least two species. However, they are often shared within certain lineages or even among evolutionary distant taxa [8]. They were first discovered in mammals [9] and subsequently identified in several groups of vertebrates and invertebrates [10,11], but their role in genome evolution remains unclear. Because of their extreme conservation, UCEs are thought to play important roles in gene regulation and development, even though UCE knockouts in mice have produced viable offspring [11]. Most of the available information on vertebrate UCEs comes from mammals and birds, but other vertebrate lineages probably display an extensive repertoire of these sequences [8,12], whose evolutionary significance could be better explored by means of comparative analyses between different taxa. Furthermore, due to their extreme conservation, UCEs are promising tools for phylogenetic inference, as shown by recent studies performed at different taxonomic levels [13,14,15,16].

In the family Lacertidae, the common lizard, *Zootoca vivipara*, shows a combination of peculiar characteristics which makes it a unique model organism for biogeographic, reproductive, genetic and chromosome studies. The common lizard has a particularly wide distribution in the Palaearctic, with several disjunct populations with alternative reproductive strategies (oviparous and viviparous) [17]. Oviparous populations belong to two genetically and geographically distant subspecies: *Z. v. louislantzi*, in the Pyrenees, and *Z. v. carniolica* (recently elevated to a full species), in the Central Eastern Alps and in the southern Carpathian Basin [6,18,19,20,21,22]. Viviparous populations are split into three taxa: the nominal subspecies (Europe, Russia, and Siberia), *Z. v. pannonica* (Slovakia, perhaps north-western Hungary, and south-eastern Austria) and *Z. v. sachalinensis* (Islands of Sakhalin and Hokkaido) [6].

The karyological features of *Z. vivipara* are also peculiar: its karyotype is of 2n = 36 chromosomes, but lacks two microchromosomes which are always present in the standard lacertid karyotype (2n = 36 telocentric macrochromosomes + two microchromosomes) [23]. Furthermore, *Z. vivipara* displays a sex chromosome system with female heterogamety [18,24,25,26,27], which has undergone extensive differentiation during the phylogenetic diversification of its various populations. The oviparous *Z. v. carniolica* and the viviparous Hungarian population of Osca, and Austrian populations, have a simple ZW sex chromosome system, with the W element shaped as a microchromosome. All the other populations have a complex Z_1_Z_2_W sex chromosome system with the W shaped as a telocentric, subtelocentric, submetacentric or metacentric macrochromosome [8,25,26].

To date, the few available studies on repetitive DNA sequences in the common lizard refer to microsatellites [20,28] and two TEs, a short interspersed element (SINE) and a degenerated gypsy-like sequence, which have a wide phylogenetic distribution in squamates [29].

In this study, we describe two newly isolated DNA sequences in the genome of *Z. vivipara*, which include two distinct truncated TEs and an UCE. We characterized the nucleotide composition, copy number, and chromosomal location of the study sequences and tested their presence in the common lizard and other vertebrate taxa by using a combination of molecular, bioinformatic and cytogenetic techniques.

## 2. Materials and Methods

Genomic DNA was extracted using the standard chloroform method [30]) from alcohol-preserved tissue samples of *Z. vivipara* specimens, representative of populations with different sex chromosome systems (ZW and Z1Z2W), different shapes of the W element, and different reproductive modalities (oviparous and viviparous) [26,31] (see Table S1). Tissue samples were from *Z. vivipara* specimens used in previous studies [18,19,20,25,26,29] (see those studies for authorities that provided research approval).

### 2.1. RAPDs Analysis

This analysis was performed on all available samples using six non-specific primers (Pharmacia) listed in Table 1.

PCR amplifications were performed in a reaction volume of 20 µL using the following conditions: 5 min at 94 °C; 40 cycles at 94 °C for 30 s; 37 °C for 2 min and 72 °C for 45 s; and 7 min at 72 °C. After electrophoresis on 1.5% agarose gel, bands from both male and female specimens were excised using the Quick gel extraction kit (Quiagen) and ligated with pGEM - T vector (pGEM-T easy vector, Promega). After transformation in DH5α cells, the inserted fragments were directly amplified via PCR using the direct T7 (5′-TAATACGACTCACTATAGGG-3′) and reverse SP6 (5′-ATTTAGGTGACACTATAG-3′) vector primers. PCR reactions were performed in 20 µL using the following conditions: 5 min at 94 °C; 36 cycles at 94 °C for 30 s; 50 °C for 30 s and 70 °C for 45 s; and, at the end, 5 min at 72 °C. Sequencing of positive colonies was performed in both directions using the BigDye Terminator kit v3.1 and an automatic sequencer ABI Prism 310 (Applied Biosystems (Waltham, MA, USA)). Chromatograms were checked and edited using CHROMAS LITE 2.3 and BIOEDIT 7.2.6.1 [32]. The newly determined sequences were submitted to GenBank (Accession numbers: OQ413073-OQ413074).

### 2.2. Quantitative Dot Blot and Fluorescence In Situ Hybridization (FISH)

Sequences obtained from amplifications of male and female positive clones were biotinylated via PCR with biotin-11-dUTP and used in quantitative dot blots and FISH analyses. PCR reactions were performed using the following conditions: 95 °C for 3 min; 30 cycles at 94 °C for 30 s; 52 °C for 30 s and 65 °C for 1 min; and, at the end, 2 min at 72 °C.

Quantitative dot blots were performed according to Mezzasalma et al. [33], starting from DNA at 200 ng (200, 100, 50, 25, 12.5, 6.25, 3.2, 1.6 ng) of male and female specimens of *Z. vivipara* from Osca, Oropa, Botany, Pourtalet, with females from Voloviz and Romany and a male from Pskov, against scalar quantities of genomic amplicons from a female specimen from Pourtalet (10, 5, 2.5,1.25, 0.62, 0.31 ng). The copy number of the isolated sequences was calculated based on the genome size of *Z. vivipara* 1.6 pg/N [34].

*Z. vivipara* chromosomes were obtained via the air-drying method as reported in Mezzasalma et al. [33] and FISH was carried out as described in [35], on metaphase plates of a female specimen from Pourtalet (see Table S1). Images were acquired through a Leica DM6000B epi-fluorescence microscope equipped with an image analysis system.

### 2.3. Phylogenetic Analysis

A phylogenetic analysis was performed using the UCE isolated in Zv817 (see Results) with conserved upstream and downstream regions up to 430 bp and including homologous sequences from representatives of the major amniote clades available from deposited WGS (see Table S2). The final alignment was generated with ClustalW in BIOEDIT 7.2.6.1 [32] and manually refined. It included homologous sequences from 55 amniote genomes with a total length of 503 nucleotide positions. Phylogenetic analysis with maximum likelihood (ML) was performed using RAxML 8.2.12 [36] under the GTRCAT model with 1000 bootstrap replicates. Bayesian inference (BI) was performed using MrBayes 3.2.7 [37] and mixed models. We ran parallel tree searches in BI over 6 million generations with four incrementally heated Markov chains (using default heating values), with a burn-in of 25% and a sampling of the chains every 1000 generations. Convergence and effective sample size (ESS) were checked using TRACER 1.5.9 [38]. Given the lack of a homologous sequence in an amniote common ancestor, the resulting tree was re-rooted on the mammal branch.

## 3. Results

### 3.1. PCR Amplification

**Figure 1:**
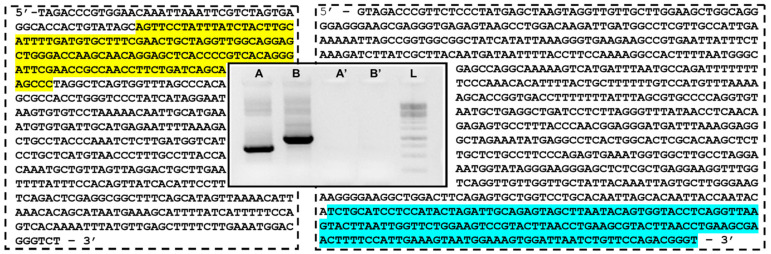
Electrophoresis on 1.5% agarose gel with RAPD PCR products using primer pair 3 (see Table 1) of male (A) and female (B) specimens of *Z. vivipara* from Pourtalet with relative blank reactions (A’ and B’) and sequences of main bands of lanes A (Zv516 on the left) and B (Zv817 on the right). L = DNA ladder (100 bp). Segments showing identity with SINE Squam1 and TC1-Mariner transposable elements are highlighted in yellow and light blue in the two sequences, respectively.

Among the six unspecific RAPD primers used, amplifications with primer pair 3 produced single-product, highly concentrated bands in both male and female specimens from Pourtalet (Figure 1). Samples from other populations showed a smeared signal up to a high molecular weight (not shown). The cloned sequences from the isolated bands produced two different consensus sequences of 516 bp (and 57.2% AT) and of 817 bp (55.8% AT), here named Zv516 and Zv817, respectively (Figure 1).

### 3.2. Bioinformatic Analysis with Zv516

A Blast search in WGS showed the presence of the whole Zv516 sequence or its fragments in the genomes of *Z. vivipara*, *Lacerta agilis*, *L. bilineata*, *L. viridis* and *Podarcis muralis nigriventris*, and retrieved a single hit covering the whole length of Zv516 in the genome of *Z. vivipara* (identity 99%) (Figure S1; Table S3). The segment from 205 bp to the 3′ end was also retrieved as a single hit in the genome of *L. bilineata*, *L. viridis* and *Podarcis muralis nigriventris*, and as two hits in *Lacerta agilis* (identity between 86% and 95%) (number of hits, e-values and identity scores are provided in Table S3, with alignments in Figure S2). Furthermore, hits of Zv516, ranging from 50 to 220 bp of the segment, were retrieved between 1800 to 5000 times in the five lacertids mentioned above (details of hit identities, e-values and score are in Table S3, with the relative alignments in Figure S3).

Searching in Repbase [39] and the BLAST nucleotide collection (n/r) with Zv516 showed that the 170 bp segment (between 50 to 220 bp) is a 5′ truncation of SINE Squam1 Non-LTR Retrotransposon with a maximum score in lacertids, namely with SINE Squam1 of the lacertid *Podarcis muralis* (score 1164; 90% similarity) and *Darevskia raddei* (AN DQ393693; max score 234; 92.94% identity) [40,41,42,43,44]. To test for the presence of the whole sequence of SINE Squam 1 in *Z*. *vivipara*, and other lacertids, the whole SINE Squam 1 sequence of *D*. *raddei* was used. The query retrieved several hits between about 1800 and >5000 (details are in Table S4).

It is noteworthy that the number of hits decreases from 23 in *Z. vivipara* to 217 in *D. valentine*, when the filters were set to Identity > 90% and Cover > 95% (see Table S4 for details and Figure S4 for the alignments). Interestingly, almost all the filtered sequences conserved Box A and B of the DNA polymerase III at their 5′ end and the short direct repeats (ACCTTT) in the 3′ end present in SINEs (Figure S4).

A WGS BLAST search with SINE squam 1 of *D*. *raddei*, limited to other families of Sauria, showed the occurrence of its segments in species of Agamidae, Dactyloidea, Phrynosomatidae, Gekkonidae and Varanidae, but with much lower coverage, e-values and scores than in Lacertidae, between two (in Gekkonidae) and 57 (in Dactyloidea) (see Table S5).

A BLAST search in the nucleotide collection archive (n/r) (using a filter set to identity > 80% and cover > 60%) with the whole max score of SINE Squam 1 of *Z. vivipara* (sequence of 351 bp as in Figure S3), produced 345 hits, and, in particular, 222 were SINE squam 1 fragments of *Podarcis muralis* and species of the genus *Darevskia*. The other hits included: seven segments of Z. vivipara mRNA (see Table S6 for the hits on enzymes and structural proteins); 83 traits of the beta-fibrinogen (FGB) gene (namely 63 of intron 7 and 20 of the beta chain) of *L*. *viridis*, *L. bilinieata and Podarcis* species; 30 of various species of *Tachydromus* (namely 24 of anonymous locus G genomic sequences and 6 of the intron of the gene RPL 13); and three of a microsatellite region of *Podarcis* species.

Searches in Refseq_rna produced the seven mRNA hits reported in the previous n/r BLAST search (Table S6).

A Query in BLAST Transcriptoma archives (TSA) of deposited lacertid sequences (Filter: coverage > 60%, identity > 90%) with the whole max score of SINE Squam 1 (351 bp) of Z. *vivipara* produced between 1 and 100 hits in *Z. vivipara*, *D. unisexualis*, *Podarcis cretensis*, *Parvilacerta parva*, *Phoenicolacerta troodoca* and *Dinarolacerta mosorensis* (details in Table S7).

Finally, a search in BLASTX with the whole SINE Squam 1 of *Z*. *vivipara* produced six protein hits (five structural and one of enzymatic protein) of *Varanus komodoensis* (four hits) and *Z. vivipara* (two hits), with a score from 44.3 to 55.8, with coverage between 20% and 43%, identity between 56.8% and 83.3%, and an e-value between 0.041 and 2 × 10^−6^ (Table S8).

### 3.3. Bioinformatic Analysis with Zv817

BLAST searches in the WGS archives of deposited lacertid sequences show that the Zv817 as a whole sequence is present as a single-copy sequence in the genome of *Z. vivipara* l, while its 5′ end (from 1 to 685 bp) is also present as a single-copy sequence in the genome of *L. bilineata*, *L. viridis* and *P. m. nigriventris*, and as two copies in the genome of *L. agilis*. Lastly, the 3′ end (from 686 to the 3′ end; using filters: 80% identity, 90% cover) is present in 504 copies in *Z. vivipara* and between 13 and 35 copies in the genome of the aforementioned lacertids (details in Table S9).

The RepBase search with the 3′ end (from 686 bp) of Zv817 shows a 70.7% identity (268 score value) with a segment of the DNA transposon Mariner-N5 PM of the sea lamprey, *Petromyzon marinus* [45,46], with several unpaired bases due to transitions (16 vs. 38) (see alignment in Figure S5).

Searching in the WGS archives of reptiles, using filters set to identity > 70% and cover > 50, with the segment of TC1 Mariner of *P. marinus*, shows its occurrence in Lacertidae (hits from 23 in *P. muralis* to 96 in *Z. vivipara*), other Sauria (hits from five in Anguimorpha to 57 in Iguania), Serpentes (hits from seven in Pythonidae to 3568 in Viperidae), and Turtles (four hits), but no hits in Crocodylia or *Sphenodon* (details in Table S10).

The BLAST search in the nucleotide collection (filters: identity > 80%, coverage > 60%) with the TC1-Mariner-like segment in *Z*. *vivipara* produced 284 hits in the *Z. vivipara* genome and 358 in the genome of the *Podarcis* species. A similar search in Rfeseq_rna of Lacertidae shows 286 and 75 hits for enzymatic and structural protein, respectively, in *Z. vivipara* and *P. muralis* genomes (details in Table S11).

Searching in the Transcriptome Sequence Archive (TSA) of deposited lacertid sequences (identity > 80%; coverage > 50%) produced 32 hits in *Z. vivipara*, three hits in *Parvilacerta parva* and one hit in *Dinarolacerta mosorensis*, for transcribed RNA sequences (Table S12). No results were produced from the search in BLASTX.

**Figure 2:**
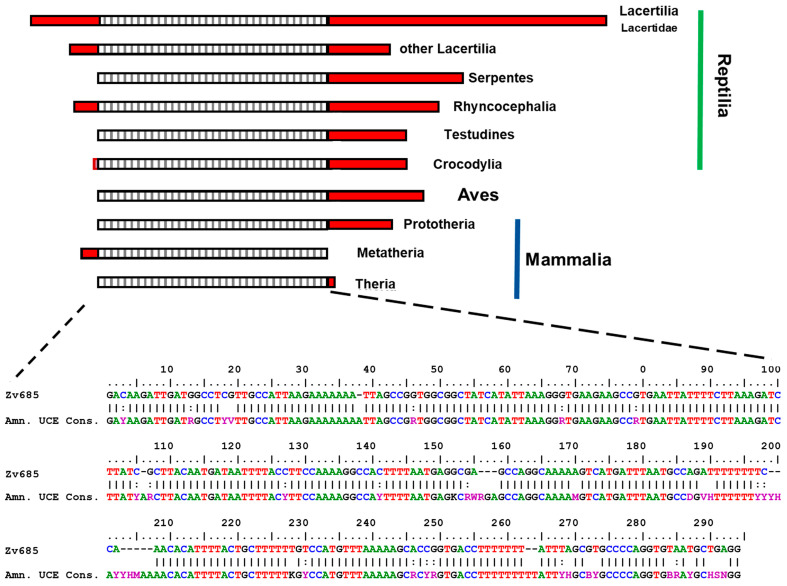
Schematic representation of the alignment of the 5′ trait (685 bp) of Zv817 from *Z*. *vivipara* with homologous sequences from amniote WGS, and below is with the amniote consensus UCE sequence. The hatched trait represents the conserved amniote UCE.

The BLAST searches with the 5′ segment (between 1 and 685 bp) of ZV817 (filters: identity > 80%; coverage > 40%) in the WGS archives retrieved one or two sequences with identity > 85% and with a progressively decreasing score, coverage and e-values, with homologous traits of species of lizards, serpents, tuatara, crocodiles, turtles, birds and mammals. The alignment of conserved traits allowed the identification of a common segment of 294 bp, showing highly homology with a UCE recently isolated in the bird genome and named UCE-3774 [15] (see Figure 2, Figure S6 and Table S2). In addition, searches in the WGS of amniote taxa with the UCE-3774 sequence here isolated show that large up and downstream regions of this element are highly conserved at family level (see Figure S7, for results in Viperidae).

### 3.4. FISH

**Figure 3:**
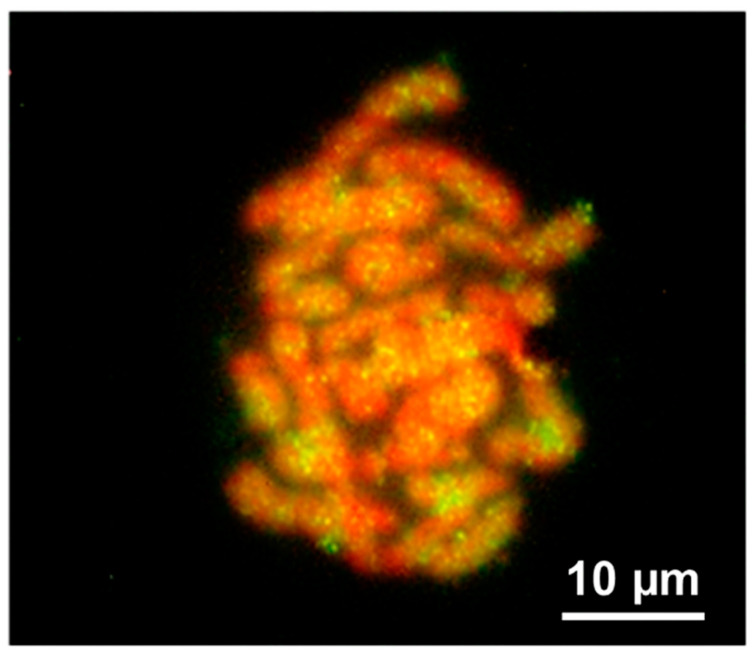
FISH with Zv561 probe on metaphase plates of female from Pourtalet (2n = 35; Z1Z2W; W telocentric).

FISH with a biotinylated probe of the Zv516 sequence shows the occurrence of interspersed hybridization signals on all chromosomes, including the W sex chromosome of female specimens of *Z. vivipara* (Figure 3) from Pourtalet (France). FISH with a biotinylated probe of Zv817 did not produce clear hybridization signals.

### 3.5. Quantitative Dot Blot

Quantitative dot blots using the biotinylated Zv516 produced hybridization signals in males and females of the studied populations of *Z*. *vivipara*. Densitometric measures of hybridization signals found that Zv516 constitutes about 0.05% of the genome of *Z*. *vivipara* (Figure S8). As will be discussed later, the hybridization signals are due the interspersed SINE Squam1 isolated in Zv516. Considering that the segments are of 170 bp (SINE Squam1) and that *Z. vivipara* has a DNA content of 1.6 pg/N [34], the resulting SINE Squam1 copy number is more than 4700 in the genome of the species.

The dot blot with the Zv817 biotinylated probe showed no or very weak hybridization signals.

### 3.6. Phylogenetic Analysis

**Figure 4:**
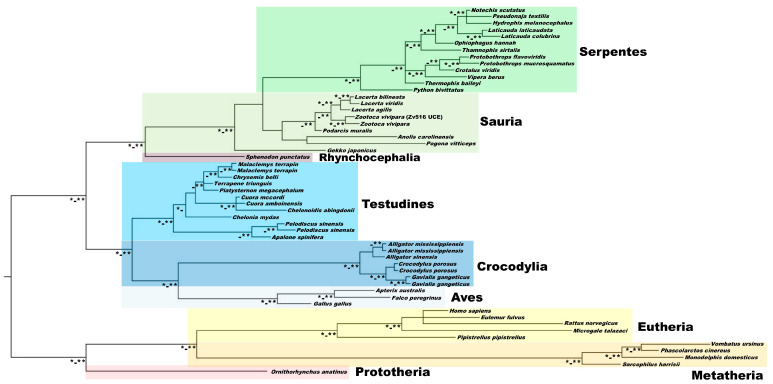
Phylogenetic tree with ML (1000 bootstrap replicates) and BI (6,000,000 generations) using the UCE isolated in Zv817 and homologous sequences from available deposited WGS. * = bootstrap values > 75; ** = Bayesian posterior support values > 0.97.

The phylogenetic analysis with the UCE-3774 included in Zv817 (see above) produced an overall and well-supported relationship with both ML and BI (Figure 4). In our phylogeny, the Prototheria are the sister group of Metatheria + Eutheria, while the relationships between diapsids support the clustering of several distinct major clades: Testudines as the sister group to Aves + Crocodylia (Archosauria), and Rynchocephalia (Sphenodon) as the sister group to Squamata.

Within Metatheria, *Sarcophilus harrisii* (Dasyuromorpha) is the sister group of a clade containing *Monodelphis domestica* (Didelphimorpha) and the sister taxon of Vombatiformes (*Phascolarctos cinereus* + *Vombatus ursinus*). In Eutheria, *Pipistrellus pipistrellus* is the sister group of a clade including *Microgale talazaci* and a tritomy including *Homo sapiens*, *Eulemur fulvus* and *Rattus norvegicus*. In the Sauropsida, the family Tryonichidae is the sister group of the other included Cryptodira, and the Alligatoridae are the sister group of Crocodylidae + Gavialidae. Within Lepidosauromorpha (Sphenodon + Squamata), *Gekko japonicus* is the sister taxon of the other squamates. *Anolis carolinensis* + *Pogona vitticeps* are the sister clade to Lacertidae, whereas the genus *Podarcis* is the sister taxon to *Lacerta* + *Zootoca*. In Serpentes, *Python bivittatus* is the sister taxon to all the other snakes, including a monophyletic clade representing Viperidae. The position of *Thermophis baileyi* is not fully resolved and *Thamnophis sirtalis* (Colubridae) is the sister group of the Elapidae.

Overall, excluding a tritomy involving *Cuora mccordi*, *C. amboinensis* and *Chelonoidis abingdonii*, the phylogenetic relationships retrieved with the UCE isolated in Zv817 are also supported at genus and species level in various phylogenetic lineages (e.g., within Crocodylia, Sauria and Serpentes).

## 4. Discussion

We identified and characterized two different genomic sequences in *Z. vivipara*, here named Zv516 and Zv817, respectively, isolated from a male and a female specimen from Pourtalet (France). Our analyses suggest that both Zv516 and Zv817 are present in the genome of both male and female *Z. vivipara* as single-copy nuclear sequences, including various truncated TEs and a UCE.

In fact, whole-length Zv516 and Zv817 sequences are present as single copies in the WGS archives of *Z. vivipara* and of various squamate species [40,44,47,48,49,50,51] (see also Tables S3 and S9). Multiple hits in different molecular databases are due to the interspersion of a SINE (SINE Squam1) and a Tc1/Mariner-like DNA transposon included in Zv516 and Zv817, respectively. The lack of direct PCR amplification of Zv516 and Zv817 in both the male and female from Pourtalet is probably due to the non-specificity of RAPD primers [52], which also produced smeared signals up to high molecular weights in all the other analyzed samples from different populations.

FISH stains and a quantitative dot blot with a Zv516 probe corroborated the bioinformatic analysis showing the presence of multiple copies (>5000 copies) in the genome of *Z. vivipara*, highlighting a significant transposition activity of the SINE Squam1

Queries in WGS of different lacertid species with SINE Squam1 retrieved > 5000 hits in *Z. vivipara*, *L. agilis*, *L. bilineata* and *D. valentini*, 4211 hits in *L. viridis*, and 1807 hits in *P. m. nigriventris* (see Tables S4 and Figure S3). It should be noted that the genomic content of other TEs varies from species to species according to class, level of activity and transposition mode [1,53]. In addition, taming of TEs in the host genome may involve copy number-dependent transposition (autoregulation) and RNA-silencing mechanisms as a specific defense mechanism against these elements [54,55].

In lacertids, SINE Squam1 and the Tc1/Mariner-like DNA transposon have different nucleotide features, transposition properties and chromosome distribution, as well as a distinct phylogenetic and evolutionary history.

In particular, the retrotransposon SINE Squam1 was first discovered in the lizards *Dareskia praticola* and *D. raddei* [41,43], and later found in the genome of varanids, iguanids, gekkonids, and snakes, and consequentially described as a squamate-specific TE [44,56]. Queries in WGS of different amniote taxonomic groups (see Results) using the SINE Squam1 segment of *Z. vivipara* allowed us to support the hypothesis of the exclusive presence of this retrotransposon in Squamata. On the other hand, SINEs have generally been found to be lineage-specific and are rarely shared among distant evolutionary lineages [57]. In contrast to most DNA markers, they can be considered nearly homoplasy free [58].

In general, SINEs are non-autonomous, non-coding Class I TEs of about 100–600 bp, which generally amplify within the host genome through RNA intermediates [59]. Active SINE Squam1 copies are of about 360 bp and are characterized by internal Box A and B of the DNA polymerase III and short direct repeats in their 3′ end [56]. The segment of SINE Squam1 isolated in this study is not an active element because it is shorter and has a partly degenerated sequence of the Box A of DNA pol III. However, queries with Squam 1 of *D. raddei* show the presence of various (multiples of ten) active SINE Squam1 sequences in lacertid species (from 29 in *Z. vivipara* to 94 in *L. viridis*; see Table S4), presenting Box A and Box B for pol III and short direct repeats in the 3′ end (see Figure S4). This evidence supports the hypothesis that squamates have few active SINE elements compared to other vertebrates, and most signs of their past transposition activities are represented by their inactive, truncated elements [60,61]. In fact, the integration of short, interspersed elements may actively modify the host DNA as a genomic parasite or as a beneficial source of genetic variability, before they are eventually tamed in the host genome [59]. Our results further indicate that SINE Squam1 probably played a significant role in the variability and evolution of the squamate genome, similarly to what has been previously documented for other similar TEs in other evolutionary lineages (see, e.g., [62]).

Unlike SINE Squam1, Tc1/Mariner is a family of cut-and-paste DNA transposons, whose structure consists of a single gene encoding a transposase enzyme flanked by inverted terminal repeats (ITRs) [62]. Tc1/Mariner-like DNA transposons usually have a much broader phylogenetic distribution than SINE retrotransposons and they have been found in invertebrates, fish, birds, mammals and squamates, while their presence in other amniotes is considered uncertain [5]. In our case, the Tc1/Mariner-like element isolated in Zv817 turned out to be similar to the DNA transposon Mariner-N5 PM of the sea lamprey, *Petromyzon marinus* [45,46], highlighting its ancient evolutionary origin and presence in vertebrates. In Squamata, this DNA transposon appears to have affected Serpentes, especially advanced caenophidian snakes, much more than Sauria (see Table S10). The segment of the Tc1/Mariner found in the present study is an inactive element, because of its shorter, highly degenerated sequence, with a truncation at its 3′ end, in particular the segment ends with the CAGAC pentamer, which is a partially degenerated ITR (CAAAC) that normally characterizes active copies of Mariner-like DNA transposon [63,64]. In fact, DNA transposons, having invaded a host genome, increase their copy number and transpose until all copies lose their activity as a result of a progressive “vertical inactivation” (see [64,65]), persisting only as truncated and inactive elements or eventually disappearing from the genome because of genetic drift [65,66].

Our results show that SINE Squam1 and Tc1/Mariner probably played a significant role in the variability and evolution of the squamate genome, similarly to what has been previously documented for other similar TEs in other evolutionary lineages (see, e.g., [66]. Queries in Refseq-rna and TSA evidenced that truncated Tc1/Mariner-like and SINE Squam 1 copies are present in mRNAs of various lacertids (see Tables S6, S7, S11, and S12).

Interestingly, we have found several SINE Squam1 remnants in intronic sequences, mostly in intron 7 of beta-fibrinogen (FGB) and RPL13 genes. In mammals, a part of the Alu-SINE element has been found in non-functional regions of introns or intergenic sequences, which could be integrated as functional modules, e.g., in producing new exons by alternative splicing, a process known as exonization [67,68,69]. Furthermore, BLAST X search shows the truncations of SINE Squam 1 have probably been integrated in some proteins of *Z. vivipara* and *V. komodoensis* (see Table S8).

The segments extra TEs, in both Zv516 and Zv817, also show peculiar nucleotide characteristics. The 3′ end of Zv516 from 218 to 516 bp was retrieved in WGS of *Z. vivipara* and other Lacertidae (identities from 94% to 98.8%), highlighting that the flanking regions of the SINE Squam 1 fragment might also be highly conserved in lacertids.

Notably, our results also showed that the segment from 48 to 345 bp of Zv817 presents a high identity (>86%) with single-copy nuclear sequences in the genome of very different vertebrate evolutionary lineages. This segment was recently isolated and identified as UCE-3774 in the bird genome [15], but to date, it has not been found in other vertebrates. Our bioinformatic and phylogenetic analyses show that UCE-3774 is absent from the amphibian and fish genome but highly conserved in all other vertebrates, allowing us to identify UCE-3774 as an amniote-conserved element. This result further supports the hypothesis of a wide appearance of UCEs during tetrapod and amniote evolution, a process possibly related to new functional, adaptive acquisitions [12].

In general, UCEs and their flanking regions are useful in phylogenetic reconstructions; however, different vertebrate clades show very different genomic amounts of UCEs and a good number of these elements are lineage-specific [9,12,14,15,70]. In our case, phylogenetic inferences with UCE-3774 among amniotes produced, with some exceptions, robust evolutionary relationships at different taxonomic levels. In our tree, Prototheria are a sister group of Metatheria and Eutheria, while within diapsids, Arcosauromorpha + turtles are a sister group to *Sphenodon* + Squamata. Among the most debated questions in vertebrate phylogeny, is the position of the turtles, which are placed alternatively within Archosauria [70], as the sister group to Archosauria [71,72], or as the sister group of Lepidosauria [73]. Interestingly, the position of turtles retrieved by our phylogenetic analysis is consistent with that reported by previous studies using more than 1000 ultra-conserved elements. However, it considers a much less inclusive taxon sample [13], and thus further supports the position of turtles as the sister group to Archosauria. Limits of our phylogeny may be found within different well-supported clades (e.g., Eutheria and Serpentes). However, this is not surprising considering the combination of limited taxon sampling and the short length of UCE-3774, which nevertheless produced a robust phylogenetic reconstruction among higher level taxa, as well as between closely related species and genera in different evolutionary lineages. It is still unknown why UCEs show such a high sequence conservation over long phylogenetic distance, but possible explanations include a combination of different evolutionary constraints, probably related to gene regulation and development (see, e.g., [74,75,76]).

We also highlight that the regions flanking UCE-3774 (up to about 1000 bp) show very high identity scores among congeneric species (>99%) and species of the same family (from 96% to 99%). These flanking regions do not show any similarity with known transcribed and/or genic regions, supporting the hypothesis that unknown functions of UCEs [9] may be shared with long traits of their flanking regions.

## 5. Conclusions

Zv516 and Zv817 are two newly isolated single-copy nuclear sequences of *Z. vivipara*. Both sequences contain a truncation of two different TEs, SINE Squam1 in Zv516 and a Tc1/Mariner-like DNA transposon in Zv817. FISH analyses showed that SINE Squam1 transposon is interspersed on all chromosomes of *Z. vivipara* and quantitative dot blot evidenced that SINE Squam1 TE is represented in the *Z. vivipara* genome with about 4700 copies. Bioinformatic analyses highlight the presence of SINE Squam1 in the genome of all squamates, while the Tc1/Mariner-like DNA transposon isolated in Zv817 was found in both invertebrates and vertebrates. Our results highlight that both TEs deeply affected the genome of different taxa, as their inactive remnants are present in the intronic region of various genes, and in the case of SINE Squam1, also in the coding region of various proteins. The regions’ extra TE in both Zv516 and Zv817 also show peculiar characteristics. In fact, in Zv516, it showed a high identity (>90%) in different Lacertidae, while the regions’ extra Tc1/Mariner of Zv817 was identified as an amniote ultra-conserved element (UCE), which produced evolutionary relationships which are consistent with a supported phylogenetic hypothesis on tetrapods.

## Figures and Tables

**Table 1:** List of non-specific primers used in PCR amplification.

Primer 1 5′-d[GGTGCGGGAA]-3′
Primer 2 5′-d[GTTTCGCTCC]-3′
Primer 3 5′-d[GTAGACCCGT]-3′
Primer 4 5′-d[AAGAGCCCGT]-3′
Primer 5 5′-d[AACGCGCAAC]-3′
Primer 6 5′-d[CCCGTCAGCA]-3′

## Data Availability

Data associated with this manuscript are available in the Supplementary Material. Newly generated DNA sequences are available on Genbank (Accession numbers: OQ413073-OQ413074).

## Supplementary Materials

Supporting information can be downloaded at: https://www.mdpi.com/article/10.3390/life13030637/s1.
