# Supplementary material for: Characterization of Two Transposable Elements and an Ultra-Conserved Element Isolated in the Genome of Zootoca vivipara (Squamata, Lacertidae)
Source: Life (Basel). 2023 Feb 24;13(3):637. doi: 10.3390/life13030637 (PMC10058329; doi:10.3390/life13030637)
Supplement: Supplementary file 1 [file life-13-00637-s001.zip › life-2149043-supplementary.pdf]

```
Score                Expect      Identities        Gaps            Strand
902 bits(1000)       00          511/517(99%)      1/517(0%)       Plus/Plus
Zv516   1          TAGACCCGTGGAACAAATTAAATTCGTCTAGTGAGGCACCACTGTATAGCAGTTCCTATT  60
                   |||||||||||||| |||||||||:|||||||||||||||||||||||||||||||||||
Z.viv   67746986   TAGACCCGTGGAACGAATTAAATTTGTCTAGTGAGGCACCACTGTATAGCAGTTCCTATT  67747045

Zv516   61         TATCTACTTGCATTTTGATGTGCTTTCGAACTGCTA-GGTTGGCAGGAGCTGGGACCAAG  119
                   |||||||||||||||||||||||||||||||||||| |||||||||||||||||||||||
Z.vivt   67747046   TATCTACTTGCATTTTGATGTGCTTTCGAACTGCTAAGGTTGGCAGGAGCTGGGACCAAG   67747105

Zv516   120        CAACAGGAGCTCACCCCGTCACAGGGATTCGAACCGCCAACCTTCTGATCAGCAAGCCCT  179
                   ||||||||||||||||||||||||||||||||||||||:|||||||||||||||||||||
Z.viv   67747106   CAACAGGAGCTCACCCCGTCACAGGGATTCGAACCGCCGACCTTCTGATCAGCAAGCCCT  67747165

Zv516   180        AGGCTCAGTGGTTTAGCCCACAGCGCCACCTGGGTCCCTATCATAGGAATAAGTGTGTCC  239
                   ||||||||||||||||||||||||||||||||||||||||||||||||||||||||||||
Z.viv   67747166   AGGCTCAGTGGTTTAGCCCACAGCGCCACCTGGGTCCCTATCATAGGAATAAGTGTGTCC  67747225

Zv516   240        TAAAAACAATTGCATGAAATGTGTGATTGCATGAGAATTTTAAAGACTGACCTACCCAAA  299
                   ||||||||||||||||||||:|||||||||||||||||||||||||||||||||||||||
Z.viv   67747226   TAAAAACAATTGCATGAAATATGTGATTGCATGAGAATTTTAAAGACTGACCTACCCAAA  67747285

Zv516   300        TCTCTTGATGGTCATCCTGCTCATGTAACCCTTTGCCTTACCACAAATGCTGTTAGTTAG  359
                   ||||||||||||||||||||||||||||||||||||||||||||||||||||||||||||
Z.vivt   67747286   TCTCTTGATGGTCATCCTGCTCATGTAACCCTTTGCCTTACCACAAATGCTGTTAGTTAG   67747345

Zv516   360        GACTGCTTGAATTTTATTTCCACAGTTATCACATTCCTTTCAGACTCGAGGCGGCTTTCA  419
                   ||||||| ||||||||||||||||||||||||||||||||||||||||||||||||||||
Z.viv   67747346   GACTGCTAGAATTTTATTTCCACAGTTATCACATTCCTTTCAGACTCGAGGCGGCTTTCA  67747405

Zv516   420        GCATAGTTAAAACATTAAAACACAGCATAATGAAAGCATTTTATCATTTTTCCAGTCACA  479
                   ||||||||||||||||||||||||||||||||||||||||||||||||||||||||||||
Z.viv   67747406   GCATAGTTAAAACATTAAAACACAGCATAATGAAAGCATTTTATCATTTTTCCAGTCACA  67747465

Zv516 480        AAATTTATGTTGAGCTTTTCTTGAAATGGACGGGTCT  516
                   |||||||||||||||||||||||||||||||||||||
Z.viv   67747466   AAATTTATGTTGAGCTTTTCTTGAAATGGACGGGTCT  67747502
```

Figure S1. Results of query in WGS archives of *Z. vivipara* with Zv516 sequence. Transitions A>G or C>T are highlighted in yellow.

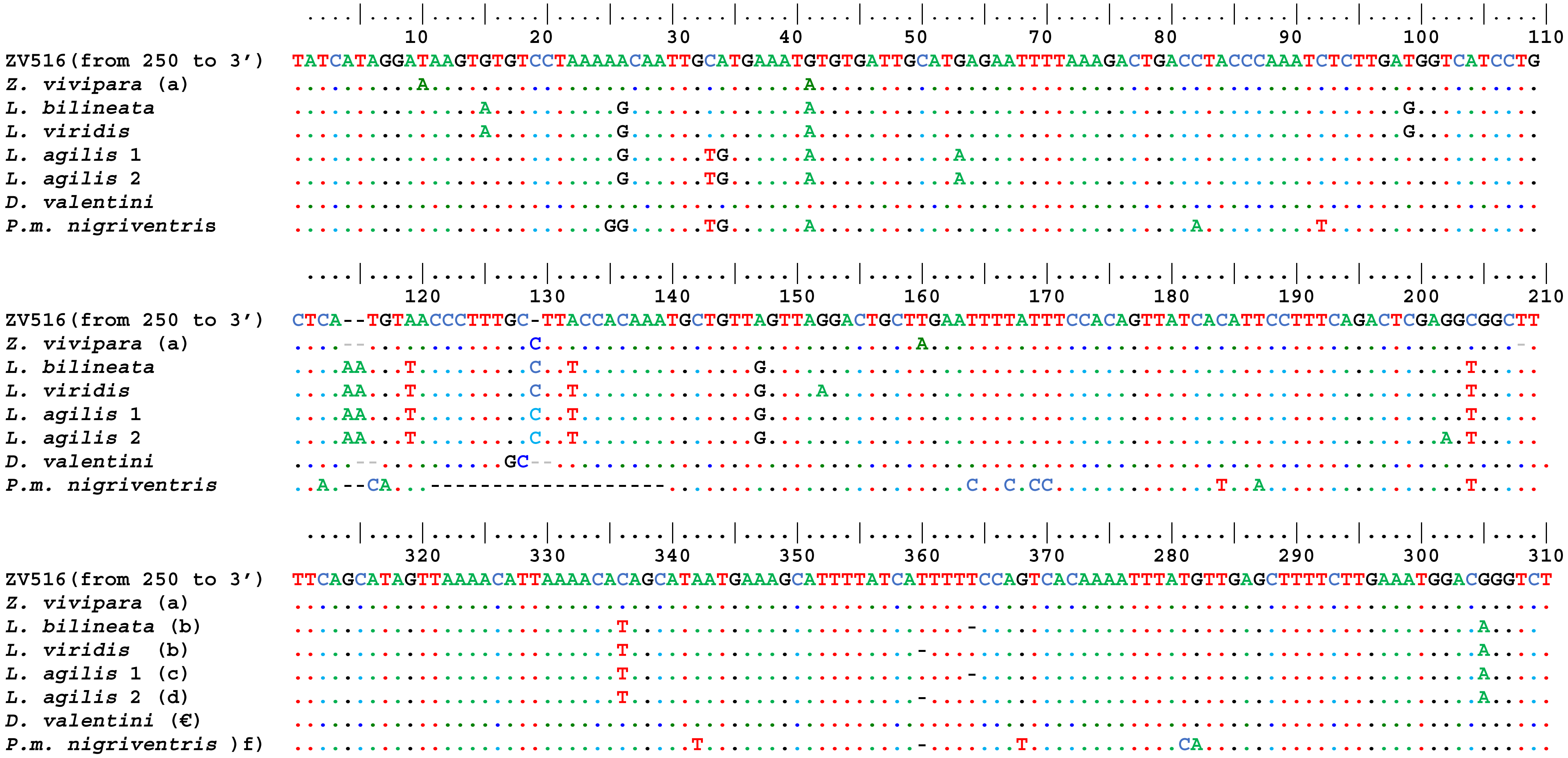

a) = JAATIO010000004 (Yurchenko et al. 2020).
b) = OFHV01006883 (Kolora 2018, unpublished)
c) = WNMS01000006 (Gemmell et al. 2019, unpliblished)
d) = WNMT01012082 (Gemmell et al. 2019, unpliblished)
e) = AIXNF010000029 (Ochkalova et al. 2022)
f) = JACVSS010000004 (Feiner et al. 2020, unpublished)

Figure S2. Alignments of the segment between 205 bp and the 3′ end of ZV516 with homologous segments with highest identities retrieved in deposited WGS archives of Lacertidae.

```
                                  10        20        30        40        50        60        70        80
                        ....|....|....|....|....|....|....|....|....|....|....|....|....|....|....|....|
Zv516 (da 50 to 229 bp) AGCAGTTCCTATTTATCTACTTGCATTTTGATGTGCTTTCGAACTGCTAGGTTGGCAGGAGCTGGGACCAAGCAACAGGA
Z.v.(JAATIO010000018)   ...G........................................................................G...
L.a.(WNMT01008209)      ...G..A..................C.....C..............................A.....TG......G...
L.b.(OFHV01005066)      ...G..A..................C.....C..............................A......G.....TG...
L.v.(OFHU01004263)      ...G..A..................C...........................................G......G...
D.v (JAIXNF010000480)   .........................C.....C.......T.............................G..........
P.m.n.(JACVSS010000)    ...G..A..................C.....C..............................A......G......G...

                                  90       100       110       120       130       140       150       160       170
                        ....|....|....|....|....|....|....|....|....|....|....|....|....|....|....|....|....|....|
Zv516 (da 50 to 229 bp) GCTCACCCCGTCACAGGGATTCGAACCGCCAACCTTCTGATCAGCAAGCCCTAGGCTCAGTGGTTTAGCCCACAGCGCCACCTGGGTCCC
Z.v.(JAATIO010000014)   ..........................................................................................
L.a.(WNMT01005274)      ............G........TC.......G...........................C........A................T.GT..
L.b.(OFHV01005066)      ........T...G.G......T........G...........................T........A......................
L.v.(OFHU01004263)      ............G.................G...........................T........A......................
D.v (JAIXNF010000480)   ............G......................................................A......................
P.m.n.(JACVSS010000)    ............G..............A..G.................T.........T........A................C.... .
```

Figure S3. Alignments of the segment between 50 to 220 bp of ZV516 with homologous segments with highest identities of WGS of *Z. vivipara* (Z.v), *L. agilis* (L.a.), *L. bilineata* (L.b.), *L.viridis* (L.v.), and *P. m. nigriventris* (P.m.n.). In brackets, the relative accession numbers. References as in Figure S2.

```
                    10        20        30        40        50        60        70        80        90       100
D. raddei   TGTCATGTTACAGTAAAAAATGGGACCCAGGTGGCGCTGTGGGTTAAACCACTGAGCCTAGGGCTTGCTG---ATCAGAAGGTCGGTGGTTCGAATCCCT
D.val  max  TGTCATGTTACAGTAAAAAATGGGACCCAGGTGGCGCTGTGGGTTAAACCACAGAGCCTAGGGCTTGCCG---ATCAGAAGGTCGGCGGTTCGAATCCTT
D.val  min  -----------------------------GGTGGCGCTGTGGGTTAAACCACAGAGCCTAGGGCTTGCCG---ATCAGAAGGTTGGTGGTTCGAATCCCC
Z.viv. max  ---------------------GGGACCCAGGTGGCGCTGT-GGTTAAACCACTGAGCCTAGGGCTTGCTG---ATCAGAAGGTCGGTGGTTCGAATTCCT
Z.viv. min  ---------------------------CAGGTGGTGCTGTGGGTTAAACCACAGAGCCTAGGGCTTGCCG---ATCAGAAGGTCGGCGGTTCGAATCCCT
L.agi. max  ---------------------GGGACCCAGGTGGCGCTGTGGGTTAAACCACAGAGCCTAGGGCTTGCTG---ATCAGAAGGTCGGCGGTTCGAATCCCT
L.agi. min  ----------------------GGACGCCGGTGGCGCTGTGGGTTAAACCACAGAGCCTAGGACTTGCTG---ATCAGAAGGTCGGTGGTTCGAATCCCT
L.vir. max  -------------TAAAGAA-GGGACGCGGGTGGCGCTGTGGGTTAAACTACAGAGCCTAGGGTTTGCTG---ATCAGAAGGTCAGTGGTTCGAATCCCT
L.vir. min  -----------------------------GGTGGCACTGTGGGTTAAACCACAGAGCCTAGGGCTTGCTG---ATCAGAAGGTTGGCGGTTCGAATCCCC
L.bil. max  --------TACAGTACCATAAGGGACCCAGGTGGCGCTGTGGGTTAAACCACAGAGCCTAGGGCTTGCTG---ATCAGAAGGGCGGCAGTTCGAATCCCT
L.bil. min  ----------------------GGACGCAGGTGGCGCTGTGGGTTAAACCACAGAGCCTAGGACTTGCTGATCATCAGAAGGTTGGCGATTCGAATCACC
P.m.n. max  ----------------------GGACGCAGGTGGCGCTGTGGGTTAAACCACAGAGCCTAGGACTTGCTG---ATCAGAAGGTCGGTGGTTCGAATCCCT
P.m.n. min  -----------------------------GGTGGCGCTGTGGGTTAAACCACAGAGTCTAGGACTTGCTG---ATCAGAAGGTTGGCGGTTTGAATCCCC
Consensus                                *****  **** ******** ** *** *****  **** *   *********   *   ** ****  *

                   110       120       130       140       150       160       170       180       190       200
D. raddei   GCGCCGGGGTGAGCTCCCGTTGCTCGGTCCCAGCTCCTGCCAACCTAGCAGTTCGAAAGCACGTCAAAGTGCAAGTAGATAAATAGGTACCGCTCTGGTG
D.val  max  GTGACGGGGTGAGCTCCCGTGGCTCGGTCCCAGCTCCTGCCAACCTAGCAGTTCGAAAGCACGTCAAAGTGCAAGTAGATAAATAGGCACCGCTCCGACG
D.val  min  GTGACGGGGTGAGCTCCCGTTGCTCGGTCCCTGCTCCTGCCAACCTAGCAGTTCGAAAGCACGTCAAAGTGCAAGTAGATAAATAGGTACCGCTGCAGCG
Z.viv. max  GTGACGGGGTGAGCTCCCGTTGCTTGGTCCCAGCTCCTGCCAACCTAGCAGTTCGAAAGCACGTCAAAGTGCAAGTAGATAAATAGGTACTGCTCCGGTG
Z.viv. min  GCAACAGGGTGAGCTCCTGTTGCTTGGTCCCAGCTCCTGCCAACCTAGCAGTTCGAAAGCACGTCAAAGTGCAAGTAGATAAATAGGTACCGCTCTGGTG
L.agi. max  GCGATGGGGTGAGCTCCCGTTGCTTGGTCCCAGCTCCTGCCCACCTATCAGTTCGAAAGCACGTCAAAGTGCAAGTAGATAAATAGGTACCGCTCTGGCG
L.agi. min  GCGACGGGGTGAGCTCAAATTGCTTGGTCCCTGCTCCTGCCCACCTAGCAGTTCGAAGGCACATCTAAATGCAAGTAGATAAATAGGTACCGCTCCAGTG
L.vir. max  GCCACGGGGTGAGCTCCCGTTGCTCGGTCCCAGCTCCTGCCCACCTAGCAGTTCGAAAGCACGTCAAAGTGCAAGTAGATAAATAGGTACCGCTCTGGCG
L.vir. min  GCGACGGGGTGAGCTCCCGTTGCTCGGTCCCAGCTCCTGCCCACCTAGCAGTTCGAAAGCATGTCAAAGTGCAAGTAGATAAATAGGTACCACTCCGGCG
L.bil. max  GCGATGGGGTGAGCTCCCGTTGCTCTGTCCCTGCTCCTGCCAAACTAGCAGTTCAAAAGCATGTCATAGTGCAAGTAGATAAAAAGGTACCGCTCCGGTG
L.bil. min  GTGACGGGGTGAGCTCCCGTTGCTCGGTCCCAGCTTCTGCCCACCTAGCAGTTCGAAAGCACGCCAAAGTGCAAGTAGATAAATAGGTACCGCTCCGG--
P.m.n. max  GCGACGGGGTGAGCTCCCGTTGCTCGGTCCCTGCTCCTGCCAACCTAGCAGTTCGAAAGCACGTCAAAGTGCAAGTAGATAAATAGGTACCGCTCCGGCA
P.m.n. min  GCGATGGGGTGAGCTCCCGTTGCTTGGTCCCTGCTCCTGCCAACCTAGCAGTTCGAAAGCACGTCAAAGTGCAAGTAGATAAATAGGCACCGCTCCGGCG
Consensus   *     **********   *****  ***** *** ***** * *** ****** ** ***   *  * ************** *** **  ***  *

                   210       220       230       240       250       260       270       280       290       300
D. raddei   GGAAGGTAAACGGCGTTTCTGTGCGCTGCTCTGGTTCGCCAGAAGCGGCTTTGTCATGCTGCCCACATGACCCGGAAGCTGTACGCCAGCTCCCTCAGCC
D.val  max  GGAAGGTAAACGGCGTTTCTGTGCGCTGCTCTGGTTCGCCAGAAGCGGCTTTGTCATGCTGGCCACATGACCTGGAAGCTGTACGCCGGCTCCCTCGGCT
D.val  min  GGAAGGTAAACGGCGTTTCCGTGCGCTGCTCTGGTTCGCCAGAAGCGGCTTAGTCATGCTGGCCACATGACCCGGAAGGTACACACCGGCTCCCTCGACC
Z.viv. max  GGAAGGTAAACGGTGTTTCTGTGTGCTGCTCTGGTTTGCCAGAAGCGGCTTTGTCATGCTGGCCACATGACCTGGAAGCTGTACGCCAGCTCCCTCAGCC
Z.viv. min  GGAAGGTAAACGGCGTTTCCGTGTGCTGCTCTGGTTCGCCAGAAGCGGCTTTGTCATGCTGGCCCCATGACCTGGAAGCTGTACGCCGGCTCCCTCAGCC
L.agi. max  GGAAGGTAAACGGCGTTTCCGTGCGCTGCTCTGGTTCGTCAGAAGCGGCTTAGTCATGCTGGCCACATGACCCAGAAGCTGTACGCCAGCTCCCTCAGCC
L.agi. min  GGAAGGTAAACGGCGTTTCCGTGCGCTGCTCTGGTTCGTCAGAAGTGGCTTAGTCATGCTGGCCACATGACCGGGAAGCTGTACGCTGGCTCCCTCAGCC
L.vir. max  GGAAGGTAAACGGCGTTTCCGTGCGCTGCTCTGGTTCGCCAGAAGTGGCTTTGTCATGCTGGCCACATGACCCGGAAGCTGTACGCCGGCTCCCTCAGCC
L.vir. min  GGAAGGTAAACAGCGTTTCCGTGCGCTGCTCTGGTTCGCCAGAAGCGGCTTTGTCATGCTGGCCACATGACCCAGAAGCTGTACGCCGGCTCCCTCAGCC
L.bil. max  GGAAGGTAAACGCCGTTTCCATGCGCTGCTCTGGTTCACCAGAAGCGGCTTTGTCATGCTGGCCACATGACCCAGAAGCTGTACGCCAGCTCCCTCAGCC
L.bil. min  -GAAGGTAAACGGCGTTTCCGTGTGCTGCTCTGGTTCACCAGAAGCGGCTTTGTCATGGTGGCCACATGACCCGGAAGCTGTACACCGGCTCCCTCAGCC
P.m.n. max  GGAAGGTAAACGGCGTTTCCGTGCGCTGCTCTGGTTCGCCAGAAGCGGCTTAGTCCTGCTGTCCACATGACCCGGAAGCTGTACGCCAGCTCCCTCAGCC
P.m.n. min  GGAAGGTAAATGGCGTTTCCGTGCACTGCTCTGGTTCGCCAGAAGCGGCTTAGTCATGCTGGCCACATGACCCGGAAGCTGTACTCCGGCTCCCTCAGCC
Consensus    *********    *****  **  ***********   ****** ***** *** ** ** ** *******  ********** *  *******  * *
```

```
                      310       320       330       340       350       360       370       380       390       400
D. raddei   AGTAACGCAAGATGAGCGCCGCAACCCCAGAGTTGGACACGACTGGACCTAATGGTAAGGGGTCCCTTTAACTTTACAGTAAAAAAT--GTCTGATTCTAGTTCA
D.val  max  AGTAACGCGAGATGAGCGCCGCAACCCCAGAGTCGGACACGACTGGACCTAATGGTAAGGGGTCCCTTTACCTTTACAGTAAAAAATATGTCTGATTCTAGTTCA
D.val  min  AATAAAGCGAGATGAGCGCCGCAA-CCCAGAGTCGGTCACGACTGGACCTAATGGTCAGGGGTCCCTTAACCTTTAC----------------------------
Z.viv. max  AATAATGCGAGATGAGCG-CGCAACCCCAGAGTCGGACACGACTGGACCTAATGGTCAGGGGTCCCTTTACCTTTAC----------------------------
Z.viv. min  AATAACGCGAGATGAGTGCTGCAACCCCAGAGTTGGTCACGACTGGACCTAATGGTCAGGGGTCTCTTTACCTTT-----------------------------
L.agi. max  AGTAAAGCGAGATGAGCGCCGCAACCCCAGAGTCGGACACAACTGGACCTAATGGTCAGGGGTCCCTTTACCTTTAC----------------------------
L.agi. min  AGTAACGTGAGATGAGTGCCGCAACCCCAGAGTCGGACATGACTGGACCTAATGGTTAGGGGTCCCTTTACCTTTA-----------------------------
L.vir. max  AGTAACGCAAGATGAG-GCCGCAACCCCAGAGTCGGACACGACTGGACCTAATGGTCAGGGGTCCCTTTACCTTTAC----------------------------
L.vir. min  ATTAACACGAGATGAGCACCGCAACTCCAGAGTTGGACACGACTGGAC---------------------------------------------------------
L.bil. max  AGTAACGCAAGATGAGCGCCGCAACCCCAGAGTCGGACACGACTGGACCTAATGGTCAGGGGTCCCTTTACCTTT------------------------------
L.bil. min  ATTAACACGAGATGAGTGCTGCAACCCCAGAATCGGATACGACTGGACCTAATGGTCAGGGGACCCTTTACCTT-------------------------------
P.m.n. max  AGTAAAGCGAGATGAGCACCGCAACCGCAGAGTCGGCCACGACTGGACCTAATGGTCAGGGGTCCCTTTACCTTTAC----------------------------
P.m.n. min  AATAAAGCGAGATGAGCGCCGCAACCCCAGAGTCGGCCACGGCTGGACCTAATGGCCAGGGGTCCCTTTACCTTTAC----------------------------
Consensus   * ***    *******    *****  **** * **  *   ******
```

Figure S4. Alignments of the SINE Squam 1 sequence AN DQ393693 of *D. raddei* with homologous sequences retrieved from BLAST queries in WGS of Lacertidae. Solid line frames include Pol III Box A and Box B; hatched lines include Short Direct Repeat. Accession numbers: *D. valentini* (max JAIXNF010000011, min JAIXNF010030895); *Z. vivipara* (max JAATIO01000000, min JAATIO010000434); *L.agilis* (max WNMS01000008, min WNMT01012604; *L. bilienata* (max OFHV01006015, min OFHV01003655); *L. viridis* (max OFHU01004678; min OFHU01004492); *P. m. nigriventris* (max JACVSS010000004, min JACVSS010000094). (References as in Figure S2).

```
query     7 CAGTGGTACCTCAGGTTAAGTACTTAATTGGTTCTGGAAGTCCGTA------CTTA-ACCTGAAGCGTAC 69
            ||||||||||||:|  || ||  |||||||||||::||||---|||------||||-||| :||::|-||
subject759 CAGTGGTACCTCGGTATACGTCGTTAATTCGTTCCAGAAG---GTAGGAGGACTTATACCGAAAATG-AC 694

query     70 TTAACCTGAAGCGAACTTTTCC-ATTGAAAGTAATGGAAAGTGGATTAATCTGTTCCAGA 128
             ||||: |||||||||:||||||||| : :||:|| ||:||: |:| |||||:||||||||
Subject 693 GTAATACCAAACGAATTTTTCCCATAAGAAATAAAGGGAAAGGAAATAATCCGTTCCAGA 634
```

Figure S5. Alignment from REPBASE of the 3′ tail (from 886 bp to 3′ end) of Zv817 of *Z. vivipara* (query) with homologous trait of the DNA transposon Mariner-N5_PM of *Petromyzon marinus* (subject). Transitions are evidenced in grey.

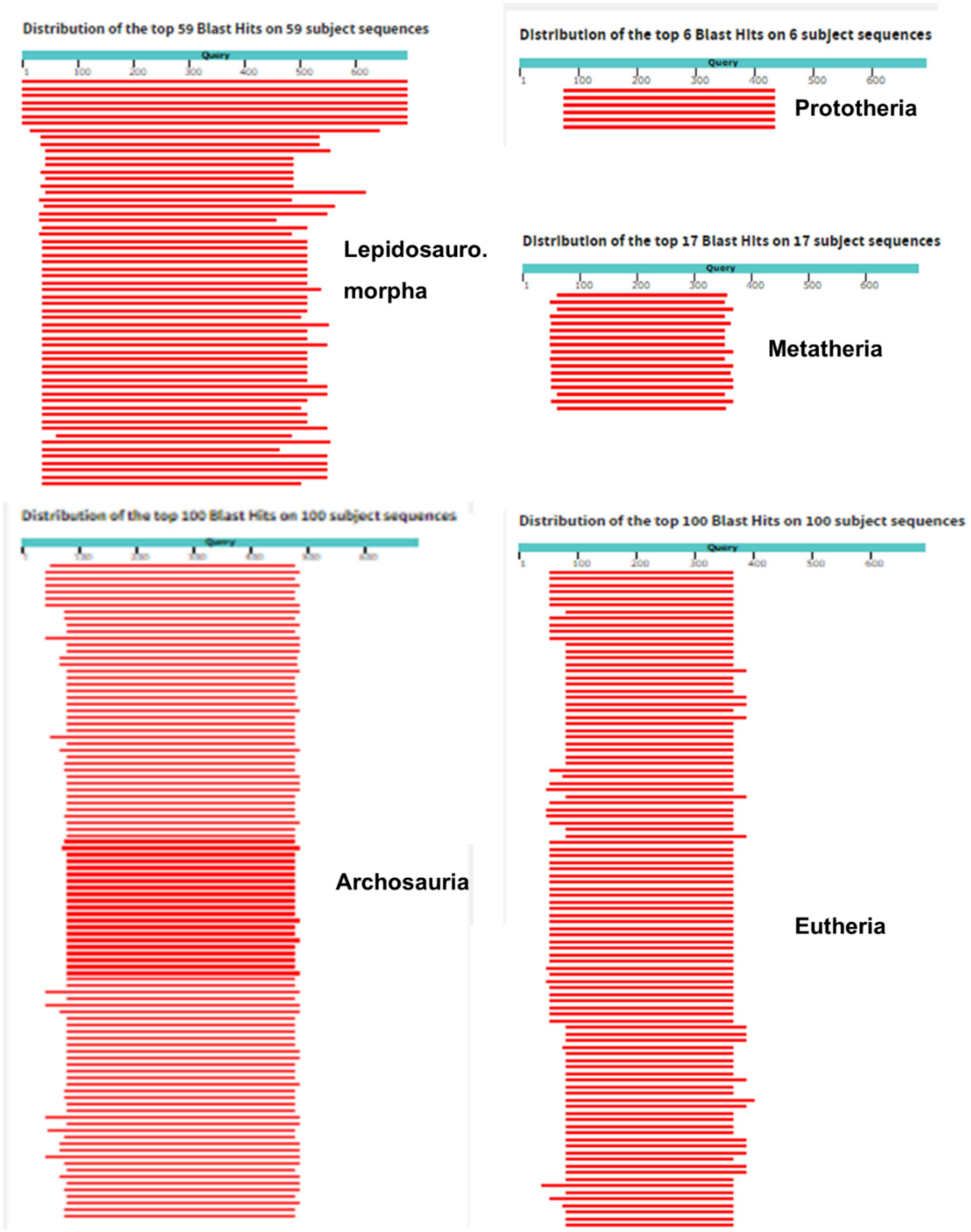

Figure S6. Graphic distribution of hits with Zv817 (from the 5′ tail to 685 bp) in Amniote nucleotide archives.

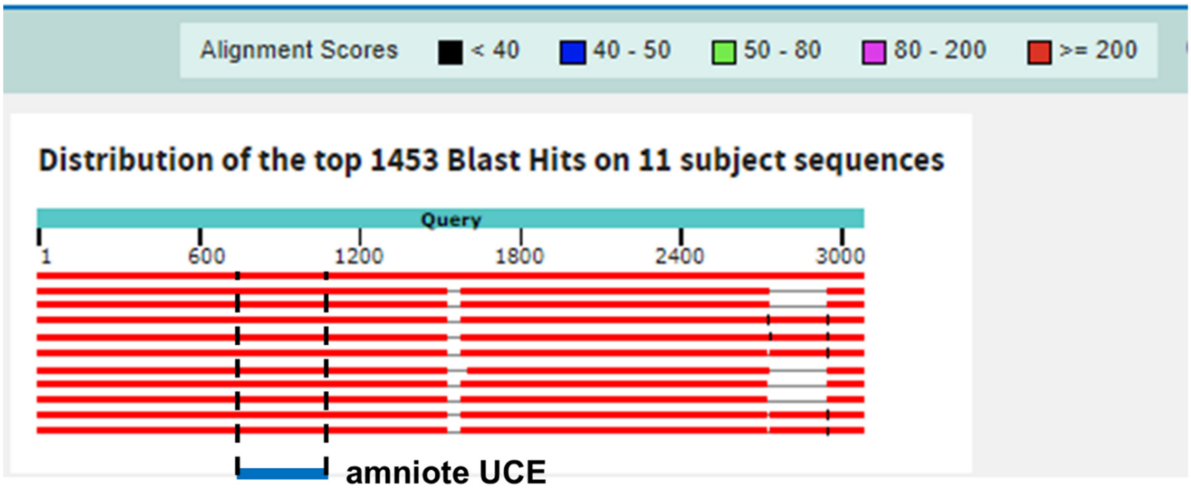

A

Description	Max Sc.	Cover	E value	Ident	Accession
Vipera berus berus isolate VBER.BE-female contig_2421, whole genome shotgun sequence	5651	100%	0.0	100.00%	JTGP01002421.1
Crotalus horridus isolate 016-059-111 Sequence_11496_17634, whole genome shotgun sequence	2303	91%	0.0	94.28%	LVCR01011496.1
Protobothrops mucrosquamatus DNA, contig: contig_17432, isolate: PMUCROS, whole genome shotgun sequence	2283	91%	0.0	94.14%	BCNE02017432.1
Crotalus viridis viridis voucher SPM297 chromosome 3, whole genome shotgun sequence	2272	98%	0.0	94.01%	PDHV02000003.1
Protobothrops flavoviridis DNA, habu1_scaffold1518, whole genome shotgun sequence	2268	98%	0.0	93.95%	BFFQ01001082.1
Crotalus adamanteus isolate Cadam-KW1264 chromosome 3 Cadam-autosomal_3100_of_8908, whole genome shotgun sequence	2266	98%	0.0	93.94%	JADPQB010005561.1
Bothrops jararaca isolate BSP84406 BJARHA_0004115, whole genome shotgun sequence	2261	90%	0.0	93.93%	JAGTXL010004108.1
Crotalus pyrrhus voucher UTA:R-60292 CMI_contig_188929, whole genome shotgun sequence	2250	91%	0.0	93.75%	JPMF01187720.1
Crotalus tigris isolate CLP2741 scf7180000021597.42775, whole genome shotgun sequence	2242	91%	0.0	93.68%	VORL01001944.1
Crotalus tigris isolate CLP2741 scf7180000021553.44426, whole genome shotgun sequence	2242	98%	0.0	93.68%	VORL01001924.1
Crotalus tigris isolate CLP2741 scf7180000017866.186403, whole genome shotgun sequence	2242	98%	0.0	93.68%	VORL01000199.1

B

Figure S7. (**A**) Schematic representation of the hits obtained with the sequence flanking amniote UCE of *Vipera aspis* (in blue light in A) in WGS archive of Viperidae and (**B**) the relative description of the obtained hits (identity and covererage set > 90%).

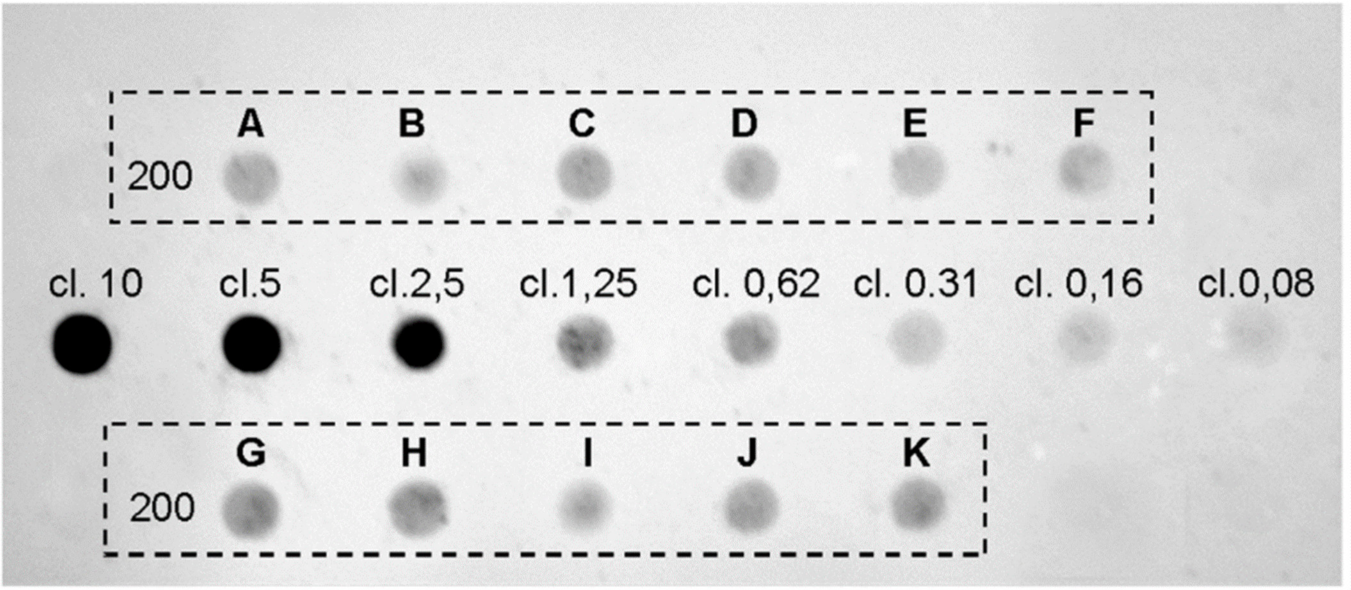

Figure S8. Quantitative dot blot with Zv516 probe to genomic DNA (200 ng) of a *Z. vivipara* samples (A-K) to scalar quantities (from 10 to 0.08 ng). Samples from: Osca (A=male, B=female); Oropa (C=male, D=female); Botany (E=male, F=female); Pourtalet (G=male, H=female); Pskov (I=male); Voloviz (J=female); Romania (K=female).

Table S1. Samples of *Z. vivipara*, with their relative clade (Surget-Groba et al., 2006), origin, sex, sex chromosome system, shape of the W chromosome and reproductive modality.

**Clade (Surget-Groba et al., 2006)**	**Origin**	**Sex**	**Sex chromosome system**	**W shape**	**Reproductive modality**
A	Oropa (Italy)	1♂, 1♀	ZZ/ZW	microchromosome	oviparous
A	Camporosso (Italy)	1♀	ZZ/ZW	microchromosome	oviparous
A	Fusine (Italy)	1♂, 1♀	ZZ/ZW	microchromosome	oviparous
B	Pourtalet (France)	1♂, 1♀	$Z_1Z_1Z_2Z_2/Z1Z_2W$	Telocentric macrochromosome	viviparous
D	Pskov (Russia)	1♂, 1♀	$Z_1Z_1Z_2Z_2/Z1Z_2W$	Telocentric macrochromosome	viviparous
D	Romany	1♀	$Z_1Z_1Z_2Z_2/Z1Z_2W$	Submetacentric macrochromosome	viviparous
E	Voloviz (Ukraine)	1♂, 1♀	$Z_1Z_1Z_2Z_2/Z1Z_2W$	Metacentric macrochromosome	viviparous
E	Botany (Slovakia)	1♂, 1♀	$Z_1Z_1Z_2Z_2/Z1Z_2W$	Metacentric macrochromosome	viviparous
F	Osca (Hungary)	1♂, 1♀	ZZ/ZW	microchromosome	viviparous

Table S2. Complete list of samples used in the phylogenetic analysis.

Sample	Accession Number	Reference
Lacerta bilineata	OFHV01000637	Kolora et al., 2019
Lacerta viridis	OFHU01002950	Kolora et al., 2019
Lacerta agilis	WNMS01000006	Gemmell et al., 2019 (unpublished)
Podarcis muralis nigroventris	JACVSS010000004	Feiner et al., 2020 (unpublished)
Pogona vitticeps	CEMB01545297	Georges et al., 2015
Zootoca vivipara	JAATIO010000004	Yurchenko et al., 2020 (unpublished)
Darevskia valentin	JAIXNF010000039	Ochkalova et al. 2022
Anolis carolinensis	AAWZ02007580	Alföldi et al., 2011
Pogona vitticeps	CEMB01545297	Georges et al., 2015
Gekko japonicus	LNDG01040291	Liu et al., 2015
Crotalus viridis	PDHV02000003	Pasquesi et al., 2018
Hydrophis cyanocinctus	RSAE01483536	Lu and Li, 2018 (unphblished)
Laticauda colubrina	BHFR01000097	Kishida et al., 2019
Laticauda laticaudata	BHFT01057486	Kishida et al., 2019
Ophiophagus hannah	AZIM01002741	Vonk et al., 2013
Python bivittatus	AEQU02037777	Castoe et al., 2011
Protobothrops flavoviridis	BFFQ01001082	Shibata et al., 2018
Protobothrops mucrosquamatus	BCNE02017432	Aird et al., 2017
Pseudonaja textilis	ULFR01000035	Edwards, 2018 (unpublished)
Thamnophis sirtalis	LFLD01011979	Warren & Wilson, 2015 (unpublished)
Thermophis baileyi	QLTV01002530	Li et al., 2018
Vipera berus	JTGP01002421	Liu et al., 2014 (unpublished)
Sphenodon punctatus	QEPC01012035	Rutherford & Gemmell, 2018 (unpublished)
Apalone spinifera	APJP01387532	Minx et al., (unpublished)
Terrapene mexicana	PRFB02000111	Deem & Warren, 2018 (Unpublished)
Chrysemys bellii	NC_024225	Shaffer et al., 2013
Platysternon megacephalum	QXTE01000205	Gong, 2019 (Unpublished)
Malaclemys terrapin	MDXI01003495	Pop et al., 2016 (Unpublished)
Malaclemys terrapin	MDXI01003563	Pop et al., 2016 (Unpublished)
Pelodiscus sinensis	AGCU01030334	Wang et al., 2013
Pelodiscus sinensis	AGCU01030335	Wang et al., 2013
Cuora amboinensis	SCPV01025202	Minx & Bermingham, 2019 (Unpublished)
Cuora mccordi	RQSS01010584	Minx & Bermingham, 2019 (Unpublished)
Chelonia mydas	AJIM01254912	Wang et al., 2013
Chelonoidis abingdonii	PKMU01007745	Quesada et al., 2018 (Unpublished)
Crocodylus porosus	MDVP01000029	Pham et al., 2016 (Unpublished)
Crocodylus porosus	JRXG02001380	Green et al., 2014
Alligator sinensis	AVPB01056796	Wan et al., 2013 ((Unpublished)
Alligator mississippiensis	LPUV01000502	Putnam et al., 2015 ((Unpublished)
Alligator mississippiensis	LPUU01002723	Putnam et al., 2015 (Unpublished)
Gavialis gangeticus	MDVQ01000044	Pham et al., 2016 (Unpublished)
Gavialis gangeticus	JRWT01216621	Green et al., 2014
Gallus gallus	PDMY01000296	Sohn et al., 2017 (unpunlished)
Apteryx haastii	PTFD01000561	Sackton et al., 2019
Falco peregrinus	MLQY01000053	Damas et al., 2016 (unpunlished)
Ornithorhynchus anatinus	NW_001783474	Warren et al., 2008
Vombatus ursinus	NW_020954603	Yazar, 2018 (unpunlished)
Sarcophilus harrisii	AFEY01099549	Miller et al., 2011
Phascolarctos cinereus	NW_018343981	Johnson et al., 2018
Monodelphis domestica	NC_008802	Mikkelsen et al., 2007

Homo sapiens	NC_000001	Gregory et al., 2006
Rattus norvegicus	NC_005104	Gregory et al., 2006
Eulemur fulvus	PVJU010000509	Johnson et al., 2018 (unpunlished)
Microgale talazaci	PVJH01030361	Johnson et al., 2018 (unpunlished)
Pipistrellus pipistrellus	PVIQ01002874	Johnson et al., 2018 (unpunlished)

Table S3. Results of Search in WGS archives of BLAST Taxid Lacertidae with Zv516. Nr.H. = number of hits; e-v = value; Id = identity; * = value of the highest hit.

Sequence		*Z. viv.*(a)*	*L. agi.*(b)*	*L. bil*(c)*	*L. vir.*(c)*	*D. val.*(d)*	*P. m. nig.*(e)*
**Zv516 (whole sequence)**	**Nr. H**	1	- - -	- - -	- - -	- - -	- - -
	**e-v**	0.0	- - -	- - -	- - -	- - -	- - -
	**id**	99%	- - -	- - -	- - -	- - -	- - -
**Zv516 (from ~50 to 220 bp)**	**Nr. H**	4854	**>5000**	**>5000**	4188	**>5000**	1810
	**e-v**	2e-80*	7e-70*	4e-57*	5e-56*	2e-124	2e-68*
	**id**	995	95%	89%	90%	93%	91%
**Zv516 (from 220 bp to 3′) 3′3′end**	**Nr. H**	1	1	1	1	1	1
	**e-v**	8e-150	4e-128	82-130	4e-128	2e.-124	1e-182
	**id**	99%	95%	95%	95%	93%	86%

a = JAATIO010000004 (Yurchenko et al., 2020) [40]; b = WNMS01000006 (Gemmell et al., 2019, unpublished); c = OFHV01006883 (Kolora et al., 2019) [41]; d = JAIXNF010000029 (Ochkalova et al. 2022) [42] e = JACVSS010000004 (Feiner et al., 2020, unpublished).

Table S4. Search with the sequence SINE Squam 1 clone 22 of *D. raddei* (AN DQ393692) in Whole Genome Sequence archives (WGS) of therein lacertid species. *, values referred to max and minus score of filtered hits.

Species	Nr Hits total	Nr Hits filtered	Score*	Coverage* (95% - 100%)	e-value*	Similarity* (90% -100%)	Accession Nr.*
Z. vivipara	>5000	23	Max 498	98%	1e-138	94.15%	JAATIO010000001<br>JAATIO010000434
			Min 435	95%	3e-120	90.15%	
L. agilis	> 5000	69	Max 520	98%	2e-145	94.33%	WNMS01000008
			Min 439	96%	5e-121	90.31%	WNMT01012604
L. viridis	4211	94	Max 511	97%	5e-143	95.12%	OFHU01004678
			Min 446	95%	3e-128	91.51%	OFHU01004492
L. bilineata	> 5000	58	Max 534	100%	1e-149	92.68%	OFHV01006015
			Min 427	97%	1e-117	90.12%	OFHV01003655
D. valentini	> 5000	217	Max 628	100%	3e-178	94,78%	JAIXNF010000011<br>JAIXNF010030895
			Min 484	95%	7e-135	91,30%	
P. muralis. nigroventris	1807	63	Max 500	100%	1e-139	93.41%	JACVSS010000004
			Min 438	97%	1e-120	91,46%	JACVSS010000094

Tabable S5. Results of WGS Blast search with SINE Squam 1 of *D. raddei* in different families of Sauria.

Family	Species	Nr Hits	Score*	Coverage* (>30%)	e-value*	Identity > 70%	Accession Nr.*
Agamidae	*Pogona vitticeps*	16	Max 135<br>Min 115	50%<br>50%	8e-30<br>1e-23	80.32%<br>78.68%	CEMB01030769<br>CEMB01025910
Dactyloidae	*Anolis carolinensis*	57	Max 141<br>Min 97	50%<br>51%	2e-31<br>4e-18	79.71%<br>80.30%	AAWZ02025084<br>AAWZ02017996
Phrynosoma-tidae	*Phrynosoma plathyrhinos*	44	Max 145<br>Min 115	30%<br>30%	6e-32<br>4e-23	89.08%<br>84.75%	JAIPUX010000439J<br>AIPUX010003289
Gekkonidae	*Gekko japonicus*	2	Max 134<br>Min 132	43%<br>42%	4e-29<br>1e-28	81.52%<br>81.87%	LNDG01099016<br>LNDG01099339
Varanidae	*Varanus komodoensis*	45	Max 134<br>Min 104	48%<br>43%	5e-29<br>4e-20	79.80%<br>78.09%	VEXN01001127<br>SJPD01000048

Table S6. Results of query in Blast Search Refseq_rna with SINE Squam 1 sequence of *Z. vivipara* (sequence 351 bp).

Description	Score	Cover	Evalue	Ident	Accession
Thiamine pyrophosphokinase1 (TPK1), transcript variant X7, mRNA	348	64%	2e-91	94.27%	XM_035101363.1
Pleckstrin homology domain containingA5 (PLEKHA5), transcript variantX12, mRNA	342	93%	1e-89	85.67%	XM_035127630.1
Plpha tubulin acetyltransferase 1(ATAT1), transcript variant X1, misc_RNA	342	68%	1e-89	92.18%	XR_004692038.1
Putative methyltransferase DDB_G0268948 (LOC118097380), transcript variant X1, mRNA	311	60%	e-80	93,36	XM_035140166.1
Katanin regulatory subunit B1 like 1 (KATNBL1), transcript variant X1, mRNA	279	62%	9e-71	89.95%	XM_035110838.1
Phosphatidylinositol glycan anchor biosynthesis class Z(PIGZ), transcript variant X3, mRNA Range 1	104	68%	6e-18	88.51%	XM_035133894.1
Phosphatidylinositol glycan anchor biosynthesis class Z(PIGZ), transcript variant X3, mRNA Range 2	95	68%	6e-18	78.30%	XM_035133894.1

Table S7. Results of BLAST search in Transcriptoma sequence archivies (TSA) of deposited sequences of lacertid species with whole SINE Squam 1 of *Z. vivipara* (hits filtered with Identity > 70% and coverage > 60%; here reported data of sequences with maximum and minimum score for each species.

**Species**	**Nr Hits**	**Score***	**Coverage* (> 60%)**	**e-value***	**Similarity* (>70%)**	**Accession Nr.**
Zootoca vivipara	18	Max 409<br>Min 283	89%<br>60%	3e-111<br>2e-73	90.19%<br>90.65%	GEHX01034844<br>GEHX01153091
Podarcis cretensis	26	Max 398<br>Min 276	79%<br>60%	6e-108<br>3e-71	92.50%<br>90.14%	GEHS01066897<br>GEHS01072124
Darevskia unisexalis	100	Max 370<br>Min 279	72%<br>60%	1e-99<br>2e-72	92.94%<br>90.23%	GEHW01278948<br>GEHW01278703
Parvilacerta parva	4	Max 364<br>Min 279	73%<br>60%	6e-98<br>2e-72	92.25%<br>90.23%	GEHM01098890<br>GEHM01098903
Phoenicola-certa troodoca	1	Max 324	70%	1e-85	90.32%	GEHR01069599
Dinarolacerta mosorensis	7	Max 303<br>Min 281	55%<br>52%	1e-79<br>2e-73	94.77%<br>94.12%	GEHQ01010225<br>GEHQ01010227

Table S8. (**A**) Results of BLASTX search in deposited Squamata sequences with whole SINE Squam 1 (351 bp) of *Z. vivipara* (*, data refer to max score alignment); (**B**) alignment with the predicted protein EYD10_ 12493 of *Varanus komodoensis.*

A

Specie - Description	Score	Cover*	E value*	Identity*	Accession*
Varanus komodoensis – Hypoth. protein EYD10_12493	54.7	34%	2e-06	67.50%	KAF7240913
Varanus komodoensis - Glut. receptor-interacting prot. 2	55.8	43%	5e-06	56.86%	KAF7249872
Varanus komodoensis - Growth fact. Recep.-bound prot.	52.4	28%	5e-05	78.79%	KAF7247830
Zootoca vivipara - Transmembrane protein 42 isoformX1	43.9	23%	0.022	70.37%	XP_034985869
Zootoca vivipara - cx9C motif-containing protein 4 isof.	44.3	20%	0.027	83.33%	XP_034969199
Varanus komodoensis - Phosphatase and actin regulator	44.3	23%	0.041	74.07%	KAF7248857

B

Hypothetical protein EYD10_12493 [*Varanus komodoensis*]

```
Query 313  GLADQKVGGSNSCDGVSSRCLVPAPANLAVRKHVKVQVDK 194
           GL D+KV GSN  +GVSSRC VPAPANLAVRKH     +K
Sbjct 55   GLVDRKVAGSNLHNGVSSRCSVPAPANLAVRKHADASREK 94
```

Table S9. Results of BLAST Search in WGS archives of deposited lacertid sequences with Zv817 (Hits filtered with identity >80% and coverage > 90%).Nr.H = number of hits; E-v = value; Id = identity; * = value of the highest hit.

**sequence**		***Z. viv.*(a)***	***L. agi*(b)***	***L. bil.*(c)***	***L. vir*(d)***	***D. val.* (e)***	***P. m. nig.*(f)***
**Zv817 (whole sequence)**	**Nr.**	1	- - -	- - -	- - -	- - -	- - -
	**e-v**	0.0	- - -	- - -	- - -	- - -	- - -
	**id**	98%	- - -	- - -	- - -	- - -	- - -
**Zv817 (from 5′ to 685 bp**	**Nr.**	1	2	1	1	1	1
	**e-v**	0.0	0.0	0.0	0.0	0.0	0.0
	**id**	99%	99%	97%%	97%	97	97%
**Zv817 (from 686 bp to 3′)**	**Nr.**	504	25	13	15	536	35
	**e-v**	2e-56	3e-48	1e-49	3e-51	1e-48	1e-51
	**id**	98%	95%	95%	96%	94%	96%

a = JAATIO010000004 (Yurchenko et al., 2020) [40]; b = WNMS01000006 (Gemmell et al., 2019, unpublished);c = OFHV01006883 (Kolora et al., 2019) [41]; d = OFHU01004521 (Kolora et al., 2019) [41]; e = JAIXNF010025682 (Ochkalova et al., 2022) [42]; f = JACVSS010000004 (Feiner et al., 2020, unpublished).

Table S10. Results of search with the trait of TC1-Mariner of *P. marinus* in Whole Genome Sequence archives (WGS) of therein reptile species. *, values refer to maximum and minimum score of filtered hits.

Species	Nr. Hits	Score*	Coverage* (50% - 100%)	e-value*	Similarity* (70% -100%)	Accession Nr.*
Z. vivipara	96	Max 68	100%	3e-09	75.00%	JAATIO010000003
		Min 44	80%	0.038	71.70%	JAATIO010000145
L. agilis	68	Max 60.8	61%	1e-07	79,22%	WNMS01000016
		Min 44.6	55%	0,038	74.29%	WNMT01000297
L. viridis	37	Max 58.1	58.1%	6e-08	78.56%	OFHU01000839
		Min 44.6	55.0%	0,038	74.29%	OFHU01000030
L. bilineata	45	Max 59.3	59.9%	2e-06	72.44%	OFHV01006511
		Min 44.6	55%	0.038	74.29%	OFHV01000090
D. valentini	76	Max 56.3	59.9%	3e-06	70,63%	OFHV01006511
		Min 42.8	55%	0.019	84.21%	OFHV01000090
P. muralis.	23	Max 56.3	100%	2e-05	71.43%	SIRZ01000013
		Min 47.3	65%	0.011	86.84%	JACVSS010000075
Anguimorpha	5	Max 54.5	68%	4e-05	85.11%	SJPD01000010
		Min 45.5	69%	0.019	81.63%	SJPD01000003
Iguania	57	Max 55.4	99%	2e-05	70.40%	CEMB01028658
		Min 44.6	96%	0.038	69.92%	CEMB01006489
Gekkota	7	Max 50	99%	2e-04	70.40%	JAHVXK010001622
		Min 46.5	76%	0.007	72,45%	JAHVXK010000809
Pythonidae	7	Max 52.7	74%	3e-05	73.40%	AEQU02240766
		Min 44.6	96%	0.038	69.92%	CEMB01006489
Elapidae	1037	Max 90.6	81%	2e-15	81.00%	SOZL01001309
		Min 42.9	74%	0.018	71.58%	AEQU02101608
Colubridae	1706	Max 98.7	80%	3e-18	83.25%	WNWU01000035
		Min 42.9	82%	0.017	71.43%	JAHWGE010095033
Viperidae	3568	Max 98.7	91.5%	4e-16	81.63%	JAGTXL010000638
		Min 44.6	79%	0.047	71.57%	BCNE02002671
Turtles	4	Max 54.5	81%	2e-04	75.00%	VOGN01020891
		Min 49.1	81%	0.010	73.53%	QXTE01000131
Crocodylia	0	-	-	-	-	-
Sphenodon	0	-	-	-	-	-

Table S11. Results of BLAST Search in Refseq_rna of deposited sequence of Lacertide with the TC1-Mariner-like segment found in *Z. vivipara*. (hits filtered with Identity > 80% and coverage >50%). *Refers to sequences with maximum score.

**Organism**	***Zootoca vivipara***	***Podarcis muralis***
**Hits**	286	75
**Score***	203	107
**Cover***	100%	618
**E-value***	6e-49	3e-20
**Identity.***	96.72%	96.72%
**Accession Number***	XM_035131993	XM_028706021
**Description***	Glycerophosphodieste phosphodiesterase 1(GDE1), transcript variantX2, mRNA	IQ motif containing E (IQCE), mRNA

Table S12. Results of BLAST Search in Transcriptoma (TSA) of deposited sequence of Lacertidae with the TC1-Mariner-like segment found in *Z. vivipara* (filter: Identity > 80%; cover >50%). * Refers to sequences with maximum score.

**Organism**	*Zootoca vivipara*	*Parvilacerta parva*	*Dinarolacerta mosorensis*
**Hits**	32	3	1
**Score***	176	99.6	89.7
**Cover***	100%	61%	61%
**E-value.***	1e-41	3e-18	1e-15
**Identity***	91.80	90.70	86.84
**Accession Nr,**	GEHX01281806	GEHM01028735	GEHQ01046318
**Description***	*Zootoca vivipara* breed wildtype contig281929, transcribed RNA sequence	*Parvilacerta parva* breed wildtype contig28751, transcribed RNA sequence	*Dinarolacerta mosorensis* breed wildtype contig46328, transcribed RNA sequence

References

Aird,S.D., Arora,J., Barua,A., Qiu,L., Terada,K. and Mikheyev,A.S. Population genomic analysis of a pitviper reveals microevolutionary forces underlying venom chemistry. *Genome Biology and Evolution* **2017**, 9: 2640-2649. doi:10.1093/gbe/evx199.

Alföldi, J.; Di Palma, F.; Grabherr, M.; Williams, C.; Kong, L.; Mauceli, E.; Russell, P.; Lowe, C. B.; Glor, R.E.; Jaffe, J.D. et al. The genome of the green anole lizard and a comparative analysis with birds and mammals. *Nature* **2011**, 477, 587-591. DOI: doi:1038/nature10390

Castoe, T.A., de Koning, J.A., Hall, K.T., Yokoyama, K.D., Gu, W., Smith, E.N., Feschotte, C., Uetz, P., Ray, D.A., Dobry, J., et al: Sequencing the genome of the Burmese python (*Python molurus bivittatus*) as a model for studying extreme adaptations in snakes. *Genome Biolology* **2011**, 12: 406. http://genomebiology.com/2011/12/7/406.

Georges, A.; Li, Q.; Lian, J.; O'Meally, D.; Deakin, J.; Wang, Z.; Zhang, P.; Fujita, M.; Patel, H. R.; et al. High coverage sequencing and annotated assembly of the genome of the Australian dragon lizard Pogona vitticeps. *GigaScience*, **2015**, 4, 45.

Green, R.E., Braun, E.L., Armstrong, J., Earl, D., Nguyen, N., Hickey, G., Vandewege, M.W. et al. Three crocodilian genomes reveal ancestral patterns of evolution among archosaurs. *Science* **2014**, 346, 1254449. doi: 10.1126/science.1254449.

Gregory, S.G., Barlow, K.F., McLay, K.E., Kaul, R., Swarbreck, D., Dunham, A., Scott, C.E. et al. The DNA sequence and biological annotation of human chromosome 1. Nature **2006**, 441: 315-321. doi: 10.1038/nature04727.

Johnson, R.N., O'Meally, D., Chen, Z., Etherington, G.J., Ho, S.Y. W., Nash, W.J., Grueber, C.E. et al. Adaptation and conservation insights from the koala genome. *Nature Genetics* **2018**, 50: 1102–1111. https://doi.org/10.1038/s41588-018-0153-5.

Kishida, T., Go, Y., Tatsumoto, S., Tatsumi, K., Kuraku, S., Toda, M. Loss of olfaction in sea snakes provides new perspectives on the aquatic adaptation of amniotes. Proceedings of Royal Society B **2019**, 286: 20191828. http://dx.doi.org/10.1098/rspb.2019.1828

Kolora, S.R.R., Weigert, A., Saffari, A., Kehr, S., Walter Costa, M,B., Spröer, C., Indrischek, H., Chintalapati, M., Lohse, K., Doose, G., et al. 2019. Divergent evolution in the genomes of closely related lacertids, *Lacerta viridis* and *L. bilineata*, and implications for speciation. *Gigascience* **2019**, 8:160. doi: 10.1093/gigascience/giy160.

Li, J-T., Gao, Y-D., Xie, L., Deng, C., Shi, P., Guan, M-L., Huang, S. et al. Comparative genomic investigation of high-elevation adaptation in ectothermic snakes. *Proceedings of the National Academy of Sciences of the United States of America* **2018**, 115: doi: 10.1073/pnas.1805348115.

Liu, Y.; Zhou, Q.; Wang, Y.; Luo, L.; Yang, J.; Yang, L.; Liu, M.; Li, Y.; Qian, T.; Zheng, Y.; et al. *Gekko japonicus* genome reveals evolution of adhesive toe pads and tail regeneration. *Nature Communications* **2015**, 6, 10033. doi: 10.1038/ncomms10033,

Mikkelsen, T., Wakefield, M.J., Aken, B., Amemiya, C.T., Chang, J.L., Duke, S., Garber, M. et al. Genome of the marsupial *Monodelphis domestica* reveals innovation in non-coding sequences. Nature 2007. 447: 167-177. doi:10.1038/nature05805.

Miller, W., Hayesb, V.M., Ratana, A., Petersenb, D.C., Wittekindta, N.E., Millerc, J., Walenzc, B. et al. Genetic diversity and population structure of the endangered marsupial *Sarcophilus harrisii* (Tasmanian devil*), Proceedings of the National Academy of Sciences of the United States of America* **2011**, 108; 12348–12353. www.pnas.org/cgi/doi/10.1073/pnas.1102838108.

Ochkalova S, Korchagin V, Vergun A, Urin A, Zilov D, Ryakhovsky S, Girnyk A, Martirosyan I, Zhernakova DV, Arakelyan M, Danielyan F, Kliver S, Brukhin V, Komissarov A, Ryskov A. First Genome of Rock Lizard Darevskia valentini

Involved in Formation of Several Parthenogenetic Species. Genes (Basel). 2022 Sep 1;13(9):1569. doi: 10.3390/genes13091569.

Pasquesi, G.I., Adams, R.H., Card, D.C., Schield, D.R., Corbin, A.B., Perry, B.W., Reyes-Velasco, J., Ruggiero, R.P., Vandewege, M.W., Shortt, J.A., Castoe, T.A. Squamate reptiles challenge paradigms of genomic repeat element evolution set by birds and mammals. *Nature Communication* **2018**, 9, 2774. https://doi.org/10.1038/s41467-018-05279-1.

Sackton. T.B., Grayson, P., Cloutier, A., Hu, Z., LiuJun S 4, Wheeler, N.E., Gardner, P.P., Clarke, J.A, Baker, A.J., Clamp, M., Edwards. S.V. Convergent regulatory evolution and loss of flight in paleognathous birds. *Science* **2019,** 364: 74-78. doi: 10.1126/science.aat7244.

Shaffer, H.B Minx, P., Warren, D.E., Shedlock, A.M., Thomson, R.C., Valenzuela, N., Abramyan, J. , et al. The Western painted turtle genome, a model for the evolution of extreme physiological adaptations in a slowly evolving lineage. *Genome Biology* **2013**, 14, R28. http://genomebiology.com/2013/14/3/R28.

Shibata, H., Chijiwa, T., Oda-Ueda, N., Nakamura, H., Yamaguchi, K., Hattori, S., Matsubara, K., Matsuda, Y., Yamashita, A., Isomoto, A, et al. The habu genome reveals accelerated evolution of venom protein genes. Scientific Reports **2018**, 81:11300. doi:10.1038/s41598-018-28749-4.

Vonk, F.J., Casewell, N.R., Henkel, C.V., Heimberg, A.M., Jansen,H.J., McCleary, R.J., Kerkkamp,H.M. et al. The king cobra genome reveals dynamic gene evolution and adaptation in the snake venom system. *Proceedings of the National Academy of Sciences of the United States of America* **2013**, 110: 20651-20656. https://doi.org/10.1073/pnas.1314702110.

Wang, Z., Pascual-Anaya, J., Zadissa, A., Li, W., Niimura, Y., Huang, Z., Li, C. et al. The draft genomes of soft-shell turtle and green sea turtle yield insights into the development and evolution of the turtle-specific body plan. *Nature Genetics* **2013**, 45: 701–706. doi:10.1038/ng.2615.

Warren, W.C., Hillier, L.W., Marshall Graves, J. A., Birney, E., Ponting, C. P., Grützner, F., Belov, K. et al. Genome analysis of the platypus reveals unique signatures of evolution. *Nature* **2008** 453: 175–183. doi:10.1038/nature06936.

Yurchenko, A.A.; Recknagel, H.; Elmer K.R. Chromosome-level assembly of the common lizard (*Zootoca vivipara*) genome. *Genome Biology and Evolution* **2020,** evaa161, https://doi.org/10.1093/gbe/evaa161.16.
